# Inflammatory Markers and Poor Outcome after Stroke: A Prospective Cohort Study and Systematic Review of Interleukin-6

**DOI:** 10.1371/journal.pmed.1000145

**Published:** 2009-09-08

**Authors:** William Whiteley, Caroline Jackson, Steff Lewis, Gordon Lowe, Ann Rumley, Peter Sandercock, Joanna Wardlaw, Martin Dennis, Cathie Sudlow

**Affiliations:** 1 Division of Clinical Neurosciences, Western General Hospital, University of Edinburgh, Edinburgh, United Kingdom; 2 Division of Cardiovascular and Medical Sciences, Royal Infirmary, University of Glasgow, Glasgow, Scotland, United Kingdom; 3 SFC Brain Imaging Research Centre, SINAPSE Collaboration, University of Edinburgh, United Kingdom; 4 Institute of Genetics and Molecular Medicine, University of Edinburgh, United Kingdom; The George Institute, Australia

## Abstract

**Background:**

The objective of this study was to determine whether: (a) markers of acute inflammation (white cell count, glucose, interleukin-6, C-reactive protein, and fibrinogen) are associated with poor outcome after stroke and (b) the addition of markers to previously validated prognostic models improves prediction of poor outcome.

**Methods and Findings:**

We prospectively recruited patients between 2002 and 2005. Clinicians assessed patients and drew blood for inflammatory markers. Patients were followed up by postal questionnaire for poor outcome (a score of>2 on the modified Rankin Scale) and death through the General Register Office (Scotland) at 6 mo. We performed a systematic review of the literature and meta-analysis of the association between interleukin-6 and poor outcome after stroke to place our study in the context of previous research. We recruited 844 patients; mortality data were available in 844 (100%) and functional outcome in 750 (89%). After appropriate adjustment, the odds ratios for the association of markers and poor outcome (comparing the upper and the lower third) were interleukin-6, 3.1 (95% CI: 1.9–5.0); C-reactive protein, 1.9 (95% CI: 1.2–3.1); fibrinogen, 1.5 (95% CI: 1.0–2.36); white cell count, 2.1 (95% CI: 1.3–3.4); and glucose 1.3 (95% CI: 0.8–2.1). The results for interleukin-6 were similar to other studies. However, the addition of inflammatory marker levels to validated prognostic models did not materially improve model discrimination, calibration, or reclassification for prediction of poor outcome after stroke.

**Conclusions:**

Raised levels of markers of the acute inflammatory response after stroke are associated with poor outcomes. However, the addition of these markers to a previously validated stroke prognostic model did not improve the prediction of poor outcome. Whether inflammatory markers are useful in prediction of recurrent stroke or other vascular events is a separate question, which requires further study.

*Please see later in the article for the Editors' Summary*

## Abstract 2

In a prospective cohort study of patient outcomes following stroke, William Whiteley and colleagues find that markers of inflammatory response are associated with poor outcomes. However, addition of these markers to existing prognostic models does not improve outcome prediction.

## Editors' Summary

**Background:**

Every year, 15 million people have a stroke. In the US alone, someone has a stroke every 40 seconds and someone dies from a stroke every 3–4 minutes. Stroke occurs when the blood supply to the brain is suddenly interrupted by a blood clot blocking a blood vessel in the brain (ischemic stroke, the commonest type of stroke) or by a blood vessel in the brain bursting (hemorrhagic stroke). Deprived of the oxygen normally carried to them by the blood, the brain cells near the blockage die. The symptoms of stroke depend on which part of the brain is damaged but include sudden weakness or paralysis along one side of the body, vision loss in one or both eyes, and confusion or trouble speaking or understanding speech. Anyone experiencing these symptoms should seek medical assistance immediately because prompt treatment can limit the damage to the brain. Risk factors for stroke include age (three-quarters of strokes occur in people over 65 years old), high blood pressure, and heart disease.

**Why Was This Study Done?:**

Many people are left with permanent disabilities after a stroke. An accurate way to predict the likely long-term outcome (prognosis) for individual patients would help clinicians manage their patients and help relatives and patients come to terms with their changed circumstances. Clinicians can get some idea of their patients' likely outcomes by assessing six simple clinical variables. These include the ability to lift both arms and awareness of the present situation. But could the inclusion of additional variables improve the predictive power of this simple prognostic model? There is some evidence that high levels in the blood of inflammatory markers (for example, interleukin-6 and C-reactive protein) are associated with poor outcomes after stroke—inflammation is the body's response to infection and to damage. In this prospective cohort study, the researchers investigate whether inflammatory markers are associated with poor outcome after stroke and whether the addition of these markers to the six-variable prognostic model improves its predictive power. Prospective cohort studies enroll a group of participants and follow their subsequent progress.

**What Did the Researchers Do and Find?:**

The researchers recruited 844 patients who had had a stroke (mainly mild ischemic strokes) in Edinburgh. Each patient was assessed soon after the stroke by a clinician and blood was taken for the measurement of inflammatory markers. Six months after the stroke, the patient or their relatives completed a postal questionnaire that assessed their progress. Information about patient deaths was obtained from the General Register Office for Scotland. Dependency on others for the activities of daily life or dying was recorded as a poor outcome. In their statistical analysis of these data, the researchers found that raised levels of several inflammatory markers increased the likelihood of a poor outcome. For example, after allowing for age and other factors, individuals with interleukin-6 levels in the upper third of the measured range were three times as likely to have a poor outcome as patients with interleukin-6 levels in the bottom third of the range. A systematic search of the literature revealed that previous studies that had looked at the potential association between interleukin-6 levels and outcome after stroke had found similar results. Finally, the researchers found that the addition of inflammatory marker levels to the six-variable prognostic model did not substantially improve its ability to predict outcome after stroke for this cohort of patients.

**What Do These Findings Mean?:**

These findings provide additional support for the idea that increased levels of inflammatory markers are associated with a poor outcome after stroke. However, because patients with infections were not excluded from the study, infection may be responsible for part of the observed association. Importantly, these findings also show that although the inclusion of inflammatory markers in the six variable prognostic model slightly improves its ability to predict outcome, the magnitude of this improvement is too small to warrant the use of these markers in routine practice. Whether the measurement of inflammatory markers might be useful in the prediction of recurrent stroke—at least a quarter of people who survive a stroke will have another one within 5 years—requires further study.

**Additional Information:**

Please access these Web sites via the online version of this summary at http://dx.doi.org/10.1371/journal.pmed.1000145.

- This study is further discussed in a *PLoS Medicine* Perspective by Len Kritharides
- The US National Institute of Neurological Disorders and Stroke provides information about all aspects of stroke (in English and Spanish); the Know Stroke site provides educational materials about stroke prevention, treatment, and rehabilitation (in English and Spanish)
- The Internet Stroke Center provides detailed information about stroke for patients, families and health professionals (in English and Spanish)
- The UK National Health Service also provides information for patients and their families about stroke (in several languages)
- MedlinePlus provides links to further resources and advice about stroke (in English and Spanish)
- The six simple variable model for prediction of death or disability after stroke is available here: http://dcnapp1.dcn.ed.ac.uk/scope/

## Introduction

A nonspecific systemic inflammatory response occurs after both ischemic and hemorrhagic stroke, either as part of the process of brain damage or in response to complications such as deep venous thrombosis. Several studies have reported that higher levels of inflammatory markers such as C-reactive protein (CRP) and interleukin-6 (IL-6) are associated with worse outcome after both ischemic [1] and hemorrhagic [2],[3] strokes. However, these studies often had methodological weaknesses, chiefly that they were too small, did not adequately adjust for confounders or assess the clinical utility of the measurements.

The addition of markers of inflammation to validated clinical prognostic models might improve the prediction of poor outcome after stroke. There are at least two validated models for predicting clinical outcome after stroke; one is based on six simple clinical variables [4] that can be applied without specific training, and the other includes the more complex National Institutes of Health Stroke Scale (NIHSS) (it measures 15 items and requires training) and age [5].

We therefore aimed to validate the suggestion that several markers of the acute phase response—CRP, IL-6, white cell count, fibrinogen, or glucose—are reliably associated with poor outcome after ischemic and hemorrhagic stroke in a large prospective cohort of stroke patients. We then wished to assess whether they improved prognostic models in the same cohort.

## Methods

### Ethics Statement

This study was conducted according to the principles expressed in the Declaration of Helsinki. The study was approved by the Lothian Research Ethics Committee. All patients or their relatives provided written informed consent for the collection of samples and subsequent analysis.

### Patients

We prospectively recruited all consenting patients with recent stroke from the emergency department; medical, neurology, and occasionally other (e.g., surgical) wards; stroke unit; and neurovascular clinics of the Western General Hospital, Edinburgh, United Kingdom, between April 2002 and May 2005 into the Edinburgh Stroke Study [6]. Clinicians recorded data at the time of assessment using a standardised structured pro forma and, in patients who consented, drew blood for measurement of inflammatory markers.

We defined a clinically definite stroke as new clinical symptoms or signs of a focal disturbance of cerebral function lasting more than 24 h of a vascular origin. We excluded patients with subarachnoid hemorrhage. At a weekly meeting, stroke physicians, neurologists, and neuroradiologists reviewed the clinical features of each patient, all brain images, and clinical progress. We defined an ischemic stroke as a clinically definite stroke in a patient whose brain imaging showed either positive evidence of a relevant ischemic lesion or was normal and excluded intracranial haemorrhage and stroke mimics. We diagnosed a stroke as an intracerebral hemorrhage if the patient's clinical features and brain imaging were consistent with acute hemorrhage. We defined pathological subtype of stroke as probably ischemic in patients with a clinically definite stroke in whom the radiological results were equivocal or unavailable and analysed them together with definite ischemic strokes. We assigned a final ischemic stroke syndrome according to the Oxford Community Stroke Project (OCSP) classification [7] based on the clinical syndrome at the time of maximum deficit modified, where appropriate, by the site and size of relevant infarcts on brain imaging. The diagnosis of stroke was made blinded to the measurement of CRP, IL-6, and fibrinogen.

### Measurement of Clinical Variables

A physician with experience in stroke medicine assessed each patient as soon as possible after presentation and recorded risk factors for stroke, current treatment, and electrocardiogram findings; measured impairment using the NIHSS [8]; and collected variables for a previously validated “six simple variables” prognostic model (age, prior dependence, able to lift both arms from the bed, able to walk without assistance, living alone at the time of the event, and orientation in time and person) [4]. We defined hypertension as a history of treated hypertension; ischemic heart disease as a history of myocardial infarction, angina, coronary artery bypass grafting, or percutaneous coronary intervention; peripheral artery disease as a history of claudication, peripheral artery intervention, or definite signs of vascular disease of the legs (e.g., absent pedal pulses); cardiac failure as definite signs of heart failure, or taking at least two medications for its treatment; and independence prior to stroke as not requiring assistance for washing, dressing, feeding, or toileting.

### Measurement of Blood Markers

Clinicians drew blood on the same day as clinical assessment or, for patients admitted to the hospital, as soon after assessment as possible. A clinical laboratory measured total white cell count (Beckman Coulter LH750 analyser) and blood glucose (Vitros Chemistry analyser). Blood samples for IL-6, CRP, and fibrinogen were transported to the laboratory on water ice, centrifuged to obtain serum and EDTA-anticoagulated plasma, and stored at −80°C until analysed. We measured CRP and fibrinogen in plasma by immunonephelometry (Prospec, Dade Behring Milton Keynes, UK) using the manufacturer's reagents and standards. We assayed IL-6 by ELISA (R & D Systems, Oxford, UK). Intra- and inter-assay coefficients of variation were 4.7% and 8.3%, 2.6% and 5.3%, and 7.5% and 8.9%, respectively. We performed all assays blind to stroke outcome.

### Assessment of Outcome

We sent each patient a validated postal questionnaire at 6 mo from his/her stroke onset date. The questionnaire measured disability with the modified Rankin Scale (mRS), a standard tool for examining outcome after stroke. We sent nonresponders a repeat questionnaire. Each patient was “flagged” at the General Register Office for Scotland, which provided information on the date and place of death. We confirmed cause of death by inspection of the relevant medical records. In primary analyses, we dichotomised a patient's outcome into “poor” if he/she was dependent on others for activities of daily living (mRS scores 3, 4, and 5) or dead, and “good” if he/she was independent in activities of daily living (mRS 0,1, and 2) 6 mo after stroke onset. In subsidiary analyses, we dichotomised patient outcome at 6 mo into alive or dead.

### Statistical Analysis

#### Association between marker levels and baseline features

In a series of bivariate analyses, we compared normally distributed baseline characteristic with Student's *t*-tests, proportions with χ^2^ tests, and positively skewed data with Wilcoxon rank sum tests. For the calculation of Pearson correlation coefficients, we logarithmically transformed positively skewed blood marker data to obtain a normal distribution. We examined the relationship between biomarker level and delay to blood taking using multivariable regression analysis.

#### Association between marker levels and outcome

We investigated the unadjusted associations between inflammatory marker level and outcome with χ^2^ for trend tests. We built a logistic regression model for the association of each inflammatory biomarker with poor or good outcome, with the terms from the previously validated six simple variable model added sequentially. We also examined logistic regression models for the association between individual biomarkers and outcome, adjusting stepwise for NIHSS, age, vascular risk factors, sex, and prior independence and living alone (domains not part of the NIHSS). For these analyses, we compared the upper and lower thirds of inflammatory marker levels for the entire sample and modelled the marker levels as linear variables. We stratified the analyses by NIHSS, OCSP, delay to blood taking, and pathological stroke type to look for evidence of effect modification.

#### Assessing the contribution of biomarkers to clinical prognostic models

We assessed the additional contribution of those inflammatory markers that were significantly associated with poor outcome after adjustment to the previously validated six simple variable model [4].

First, we assessed whether blood markers improved the goodness of fit of existing models using the likelihood ratio statistic. Second, to compare the ability of models to discriminate between good and poor outcome, we calculated areas under receiver operator curves (AUC). An AUC of 1 indicates perfect discrimination and 0.5 no discrimination. Third, we assessed calibration (whether the average predicted risk of poor outcome in subgroups matches that observed in the cohort) with the Hosmer Lemeshow χ^2^ statistic. Fourth, we assessed the ability of the best performing model including biomarkers to one without by examining risk stratification tables [9]. We used the methods of Pencina et al. [10] to calculate net reclassification improvement (NRI). NRI is a measure that takes into account the correct movement of individuals between categories of predicted risk (i.e., numbers moving correctly or incorrectly up or down) to estimate overall improvement. We prespecified thresholds of<10% and>90% for predicted probability of poor outcome as we believe that one would need to be very certain of a good or poor outcome before avoiding treatments such as thrombolysis or selecting patients for palliative care only.

All *p* values reported are two-sided and we considered *p*<0.05 statistically significant. We performed statistical analyses with Stata (Version 10.1, College Station, TX, USA).

### Systematic Review

We searched Medline and EMBASE from 1966 to December 2008 for studies in patients with acute stroke that measured IL-6 and assessed clinical outcome. The search strategy included 13 terms for ischemic stroke and two for IL-6. Prognostic studies were identified using high-sensitivity search terms [11], together with common outcome measurements from stroke research (Rankin, NIHSS, Glasgow outcome scale) (see MOOSE checklist, Text S1). We included studies if they (a) reported results for patients with acute stroke (not transient ischemic attack); (b) assayed a venous IL-6 in stroke patients; (c) measured outcome using death, disability, or handicap scales; and (d) reported results in a manner that allowed calculation of OR for poor outcome or death per unit increase in marker to allow comparison of measures of association between studies. The result of the literature search is available on request from the authors. We extracted data from logistic regression models reporting the association between IL-6 and poor outcome or death after stroke, and the degree of adjustment for age, stroke severity, and other potential confounders. We performed fixed effects meta-analysis with Stats Direct Version 2.7.2.

We prepared this paper with reference to the STROBE [12] guidelines for reports of observational epidemiological studies, the REMARK [13] guidelines for reports of prognostic variables, and the MOOSE [14] guidelines for the meta-analysis of observational studies.

## Results

### Baseline Characteristics

**Figure 1:**
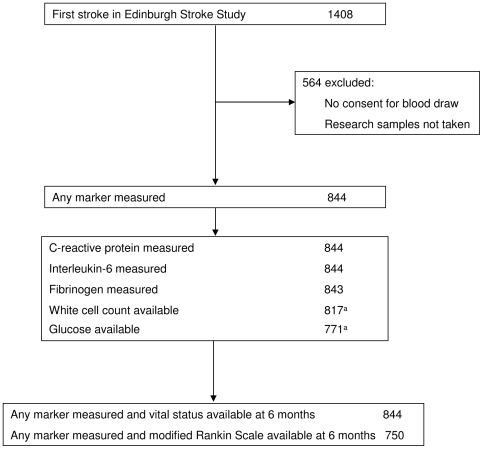
Flowchart of data available in the study. ^a^Results are incomplete for glucose and white cell count, as for outpatients these results were sometimes reported to the general practice rather than the central results database.

We assessed 1,408 patients, of whom 844 (60%) had blood drawn for markers of inflammation. Of these, 785 (93%) had a definite ischemic stroke, 16 (2%) a probable ischemic stroke, and 43 (5%) a hemorrhagic stroke. Those included were similar to those who were not, in age, sex, and the proportions with hypertension, peripheral, or cardiac vascular disease; diabetes; or atrial fibrillation. On average, compared to those without biomarker data, patients with biomarker data had milder strokes (median NIHSS 1 versus 2, *p*<0.001; proportion total anterior circulation stroke syndrome 7.7% versus 14.7%, *p* = 0.001, respectively), as patients admitted to the hospital and those with more severe symptoms were less likely to be recruited because of practical barriers to obtaining and processing research blood samples and obtaining informed consent or assent [6]. Included patients were also less likely to have a diagnosis of cardiac failure (4.3% versus 8.3%, *p* = 0.002). The median delay from stroke to blood taking was 13 d (IQR 6 to 22 d). Of those patients who had blood drawn for blood markers, 6-mo mRS data were available in 750/844 (89%) and vital status at 6 mo was available in all patients. At 6 mo, of the 844 patients, 59 were dead and 238 were dead or disabled. Data completeness is summarised in Figure 1. Deaths were due to the initial or recurrent stroke (35/59, 59%); vascular disease of the heart, legs, or bowel (9/59, 15%); cardiac failure (5/59, 9%); cancer (5/59, 9%); and bowel perforation, chronic obstructive pulmonary disease, or pneumonia (5/59, 8%).

For all markers there was a weak though statistically significant (*p*<0.001) negative relationship between the natural logarithm of marker and time. The Pearson correlation coefficients for the relationship between time in days and the natural logarithm of each marker were: glucose, *r* = −0.07; white cell count, *r* = −0.12; fibrinogen, *r* = −0.12; CRP, *r* = −0.14; and IL-6, *r* = −0.19. In multivariate regression models with time as the independent variable, after adjustment for age and stroke severity measured by NIHSS, these relationships were even weaker and not statistically significant.

Table 1 summarises the baseline data for all those patients from whom blood was drawn for markers and for those with good and poor outcome at 6 mo. Patients who died or had poor outcome were older; had more severe strokes; and had more ischemic heart disease, previous strokes or transient ischemic attacks, diabetes, congestive cardiac failure, and atrial fibrillation. They were more likely at the time of stroke to live alone, be dependent on others for activities of daily living, be disorientated, have arm weakness, and be unable to walk. They had higher levels of IL-6, CRP, fibrinogen, white cell count, and glucose.

**Table 1:** Baseline characteristics of biomarker cohort and their association with death and poor outcome.

Characteristic	Total Cohort (*n* = 844)	Good Outcome at 6 mo (mRS = 0,1,2) (*n* = 512)	Poor outcome at 6 mo (mRS = 3,4,5 or dead) (*n* = 238)	*p*-Value
Age, mean (SD)	72 (11)	70 (11)	75 (11)	<0.001^a^
Male sex, number (%)	445 (53)	275 (54)	115 (48)	0.169^b^
NIHSS^d^, median (IQR)^e^	1 (4)	1 (2)	4 (7)	<0.001^c^
**Laboratory measurements, median (IQR)**				
Interleukin-6 (pg/ml)	4.0 (4.8)	3.3 (3.2)	6.1 (7.5)	<0.0001^c^
C-reactive protein (mg/l)	3.4 (8.1)	2.6 (5.7)	7.1 (18.8)	<0.0001^c^
Fibrinogen (g/l)	4.5 (1.6)	4.3 (1.4)	5.0 (1.9)	<0.0001^c^
White cell count (×10^9^/l)^f^	8.0 (3.1)	7.7 (2.9)	8.5 (3.1)	<0.0001^c^
Glucose(mmol/l)^g^	5.6 (1.9)	5.5 (1.7)	6.0 (2.1)	0.0002^c^
Cholesterol (mmol/l), mean (SD)	5.2 (1.3)	5.2 (1.2)	5.1 (1.3)	0.189^a^
**Pathological stroke type, number (%)**				
Definite ischemic stroke	785 (93)	484 (95)	215 (90)	0.006^b^
Definite hemorrhagic stroke	43 (5)	18 (4)	21 (9)	
Probable ischemic stroke	16 (2)	10 (2)	2 (1)	
**OCSP ischemic stroke syndrome number (%)**				
Total anterior circulation infarction	53 (7)	10 (2)	32 (15)	<0.001^b^
Partial anterior circulation infarction	352 (44)	225 (46)	96 (44)	
Lacunar infarction	221 (28)	143 (29)	53 (24)	
Posterior circulation infarction	124 (16)	80 (16)	28 (13)	
Unclassified	51 (6)	36 (7)	8 (4)	
**Six simple variable model** h **number (%)**				
Living alone	324 (38)	327 (36)	105/237 (44)	0.033^b^
Independent pre-stroke	799 (95)	502 (98)	209 (88)	<0.001^b^
Normal verbal Glasgow coma scale	754 (90)	492/509 (97)	185/237 (78)	<0.001^b^
Able to lift both arms	749 (89)	494/511 (97)	180 (76)	<0.001^b^
Able to walk	640 (76)	464/511 (91)	117 (49)	<0.001^b^
**Stroke risk factors number (%)**				
History of hypertension	453 (54)	244 (52)	143 (60)	0.047 b
Prior ischemic heart disease	234 (28)	125 (24)	86 (36)	0.001^b^
History of diabetes	103 (12)	52 (10)	41 (17)	0.006^b^
History of peripheral vascular disease	36 (8)	40 (8)	18/235 (8)	0.941^b^
History of cardiac failure	40 (5)	11/511 (2)	25/237 (11)	<0.001^b^
Atrial fibrillation (previous or current)	162 (19)	73 (14)	69 (29)	<0.001^b^
Prior stroke or transient ischemic attack	262 (31)	144 (28)	86 (36)	0.027^b^
Smoker (current or within 1 y)	275/829 (31)	163/508 (32)	73/232 (31)	0.886^b^

a *t*-test.

b χ^2^ test.

c National Institute of Health Stroke Scale.

d 482 good outcome and 224 poor outcome strokes.

e Wilcoxon rank sum test.

f 496 good outcome and 233 poor outcome strokes.

g 471 good outcome and 218 poor outcome strokes.

h The sixth variable in this model is age.

### Relation of Markers to Outcome with and without Adjustment for Other Factors

**Figure 2:**
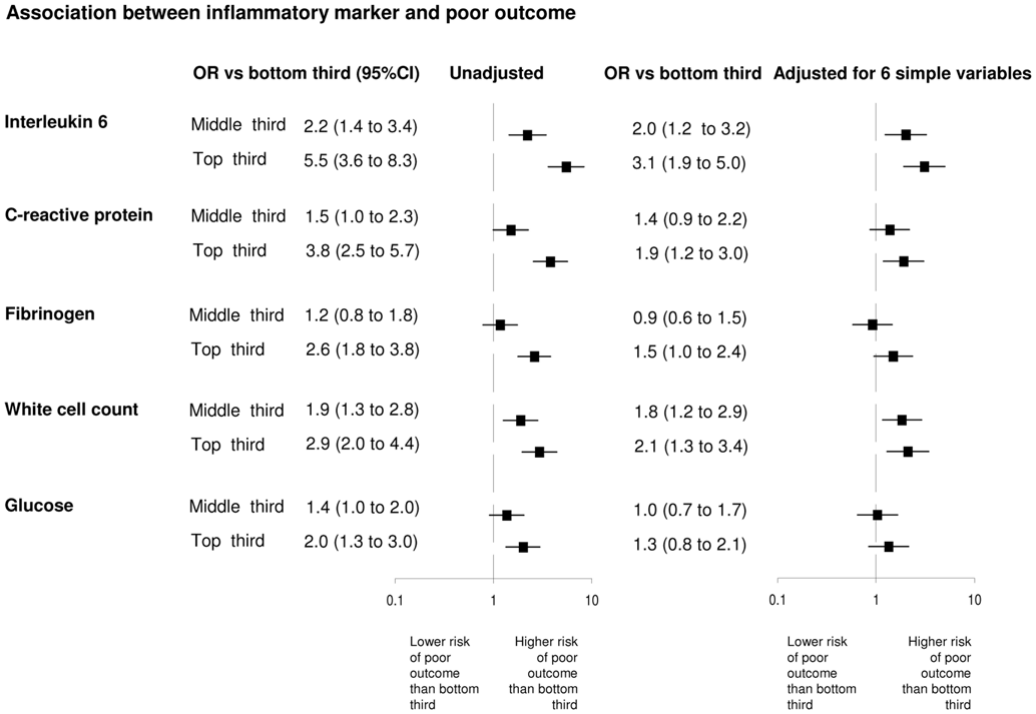
Association between levels of inflammatory marker versus poor outcome (mRS>2 or death). Expressed as ratio of odds in middle and top thirds of marker distribution, versus the referent lower third. Dotted line indicates OR = 1 (i.e., same odds as lower third). ORs are reported unadjusted and adjusted for six simple variables (age, living alone, independent of activities of daily living prior to stroke, normal verbal Glasgow coma scale, able to lift arms from bed, able to walk). Tertiles of IL-6: 2.8 and 5.5 pg/l; CRP: 1.9 and 7.1 mg/l; fibrinogen: 4.1 and 5.1 g/l; white cell count: 7.0 and 9.1×10^9^ cells/l; and glucose: 5.2 and 6.3 mmol/l.

**Figure 3:**
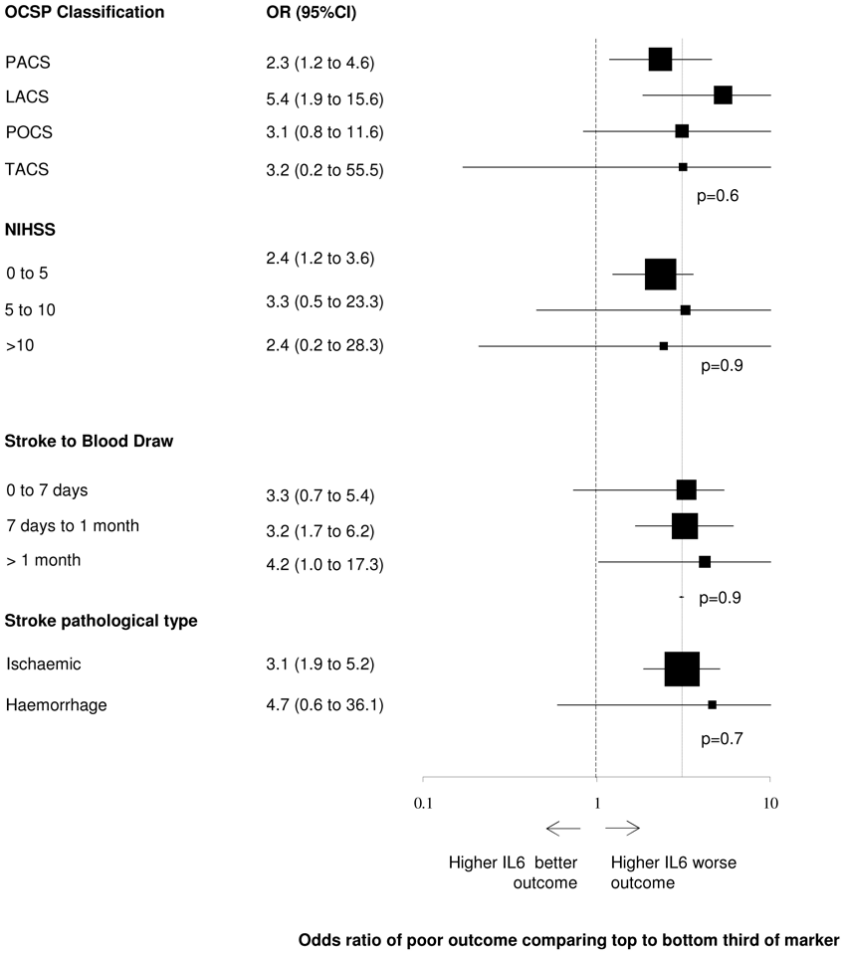
Association between upper third and lower third of IL-6 by subgroups. Each OR is adjusted for the six simple variables (age, living alone, independent of activities of daily living prior to stroke, normal verbal GCS, able to lift arms from bed, able to walk), and the estimate for the whole cohort is given by the vertical dashed line. OR of>1 indicates that increased levels of marker are associated with poorer outcome in that category of patient. The *p* values are derived from tests for heterogeneity. LACS, lacunar stroke syndrome; PACS, partial anterior strokesyndrome; POCS, posterior circulation stroke syndrome; TACS,total anterior stroke syndrome.

There were strong positive associations between marker levels and the odds of poor outcome (Figure 2). The risk of poor outcome rose by each third of IL-6 (χ^2^ trend *p*<0.001), CRP (χ^2^ trend *p*<0.001), fibrinogen (χ^2^ trend *p*<0.001), white cell count (χ^2^ trend *p* = 0.002), and glucose (χ^2^ trend *p* = 0.001). The risk of death also rose by each third of marker (χ^2^ trend *p*<0.001 for each marker) (unpublished data), though in general the association between marker thirds and death was stronger than for poor outcome. After adjustment for age, and at the onset of stroke, whether the patient lived alone, was independent of activities of daily living, was orientated, was able to lift his/her arms or walk, the odds ratios were attenuated for the association with poor outcome (top versus bottom third: IL-6, OR: 3.1, 95% CI: 1.9–5.0; CRP, OR: 1.9, 95% CI: 1.2–3.1; fibrinogen, OR: 1.5, 95% CI: 1.0–2.4; white cell count, OR: 2.1, 95% CI: 1.3–3.4; and glucose OR: 1.3, 95%, CI: 0.8–2.1) and death (unpublished data). Adjustment for the association between marker levels and poor outcome for NIHSS, age, vascular risk factors, sex, and prior independence and living alone led to only minor changes in the magnitude of these odds ratios for the association with poor outcome. After additional adjustment for other markers, only the association between IL-6 and poor outcome remained independently significant (OR: 2.4, 95% CI: 1.3–4.5). Further adjustment of the associations with death was not performed because of the relatively small number of events. There was no material difference in the magnitude, direction, or significance of the association between IL-6, CRP, and white cell count (data shown for IL-6) and outcome after stratifying the analysis by stroke subtype, stroke severity, clinical stroke syndrome, or delay to blood taking after stroke (Figure 3).

The crude increase in the odds of death or disability per unit increase in marker level was lowest for CRP and highest for fibrinogen, though the range of the fibrinogen (1.2–9.6 g/l) was smaller than CRP (0.159–263 mg/l). After adjustment for the six simple variables, the associations between IL-6, CRP, and white cell count remained statistically significant (Table 2).

**Table 2:** The association between marker levels and poor outcome after stroke.

Markers	Odds Ratio Per Unit Increase in Marker Level (95% CI)		
	Unadjusted Estimate	Adjusted for Six Simple Variable	Adjusted for NIHSS, Age, Risk Factors for Recurrent Stroke^a^, Living Alone, and Prior Independence
IL-6 (pg/ml)	1.14 (1.10–1.17)	1.07 (1.03–1.11)	1.05 (1.01–1.09)
CRP (mg/l)	1.02 (1.01–1.03)	1.01 (1.00–1.01)	1.01 (1.00–1.01)
Fibrinogen (g/l)	1.35 (1.21–1.51)	1.12 (0.98–1.28)	1.05 (0.90–1.21)
White cell count (×10^9^/l)	1.14 (1.08–1.21)	1.08 (1.01–1.16)	1.06 (0.99–1.14)
Glucose (mmol/l)	1.06 (1.00–1.12)	1.04 (0.97–1.12)	0.96 (0.87–1.05)

a Previous diabetes, history of cardiovascular disease, history of peripheral vascular disease, history of cardiac failure, history of hypertension, current or history of atrial fibrillation.

### Does the Addition of Marker Data Improve the Utility of Clinical Predictive Models?

We added data for markers with independent associations with poor outcome (IL-6, CRP, and white cell count) as continuous variables to the previously validated six simple variable model (Table 3) [4]. Model fit was improved significantly after the addition of IL-6 or white cell count, though not CRP. Model calibration was adequate after the addition of IL-6, white cell count, and CRP. However, AUC was improved significantly only after the addition of IL-6 to the six simple variable model, though not after the addition of white cell count or CRP alone. A model with the six simple variables and all of the inflammatory markers was well calibrated but had a similar AUC to a model with the six simple variables and IL-6 alone (*p* = 0.8). As the NIHSS and age model was poorly calibrated in this cohort (Hosmer-Lemeshow χ^2^ *p* = 0.01), it was not examined further.

**Table 3:** Performance of models to predict poor outcome after stroke.

Model	Likelihood Ratio Statistic	*p*-Value^a^	Hosmer-Lemeshow χ^2^ (Estimate of Model Calibration)	*p*-Value^b^	AUC (95% CI)	*p* c
**1. Six simple variables**	**Reference**	**Reference**	**6.2**	**0.63**	**0.78 (0.74–0.83)**	**Reference**
2. Six simple variables+IL-6	10.9	<0.01	8.0	0.43	0.80 (0.76–0.84)	<0.01
3. Six simple variables+CRP	3.4	0.06	6.7	0.57	0.78 (0.75–0.82)	0.09
4. Six simple variables+white cell count	5.62	0.02	3.3	0.91	0.78 (0.74–0.82)	0.53
5. Six simple variables+white cell count+CRP+IL-6	13.39	<0.01	12.0	0.15	0.80 (0.76–0.83)	0.01

Performance of six simple variables model (age, living alone, independent of activities of daily living prior to stroke, normal verbal GCS, able to lift arms from bed, able to walk) and addition of IL-6, CRP, and white cell count as continuous variables.

a The likelihood ratio test compares a goodness of fit between models with and without biomarker data. *p*<0.05 indicates that the model with biomarkers gives a significantly better fit of the data.

b The Hosmer Lemeshow test compares the observed number of people with events to that predicted by the model. *p*>0.05 indicates that the model is well calibrated.

c AUC = 1 indicates perfect discrimination of a model between patients with good and bad outcomes. *p*<0.05 indicates that the model containing biomarkers has a significantly higher AUC than one without.

We compared the proportions of patients with predicted high (>90%) and low (<10%) risks of poor outcome by the six simple variable model with and without the addition of IL-6 (Table 4). The addition of IL-6 to the six simple variable model increased the proportion of patients in the lowest risk category from 2.5% to 4.4% and the proportion in the highest risk category from 2.2% to 3.0%; that is, an extra 2.6% (95% CI: 1.7–4.1) were moved from indeterminate (10%–90%) to determinate categories (>90% or<10%). The models correctly classified those in the highest risk category as having a poor outcome, in 91% (95% CI: 73–98) of patients for the model including IL-6, and 94% (95% CI: 73–99) for the model without. The models incorrectly classified patients in the lowest risk category in 12% (95% CI: 5%–27%) for the model including IL-6 and 16% (95% CI: 6%–38%) for the model with the six simple variables alone. The NRI after the addition of IL-6 to the six simple variable model (5%, *p* = 0.014) was small.

**Table 4:** Risk stratification tables to assess the clinical significance of added predictive value of IL-6 to the six simple variable model.

Predicted Risk of Poor Outcome from Six Simple Variable Model Alone	Predicted Risk of Poor Outcome from Six Simple Variable Model with IL- 6				Total % Reclassified
	<10%	10%–50%	50%–90%	>90%	
**<10%**					
Patients (*n*)	14	5	—	—	—
% reclassified	—	26	—	—	26
Observed % poor outcome	14	20	—	—	—
**10%–50%**					
Patients (*n*)	19	534	4	—	—
% reclassified	3	—	7	—	4
Observed % poor outcome	11	20	75	—	—
**50%–90%**					
Patients (*n*)	—	4	137	10	-
% reclassified	—	3	—	7	9
Observed % poor outcome	—	50	69	90	-
**>90%**					
Patients (*n*)	—	—	4	13	—
% reclassified	—	—	23	—	23
Observed % poor outcome	—	—	100	92	—
**Total**					
Patients (*n*)	33	543	145	23	—
Observed % poor outcome	12	20	70	91	—

### Systematic Review and Meta-Analysis

**Figure 4:**
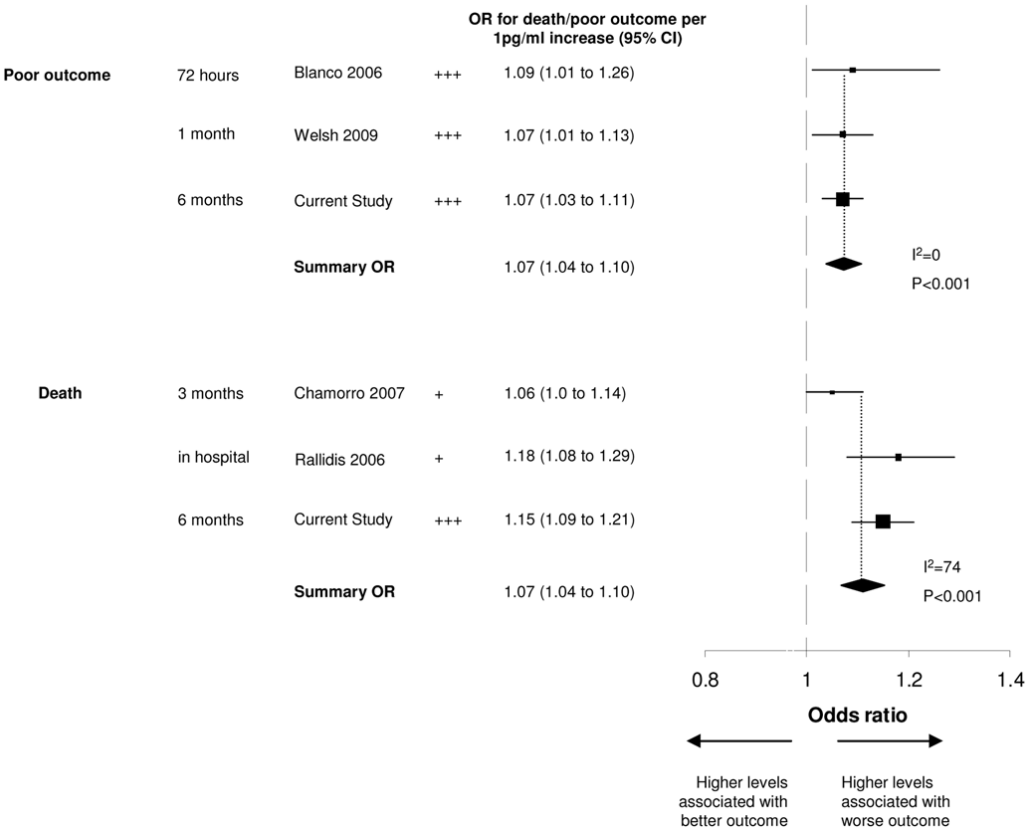
Systematic review and meta-analysis of studies of IL-6 with available data. OR for death or poor outcome is presented per unit increase in marker levels. Sizes of squares are proportional to the number of patients in each study. Summary estimates are calculated by fixed effects meta-analysis. The *p* values show statistical significance of summary estimate of effect, and I^2^ is reported as a measure of heterogeneity between studies used to calculate the summary OR. + = adjusted for age or stroke severity; ++ = adjusted for age and stroke severity; +++ = adjusted for age, stroke severity, and other factors.

The literature search identified 146 studies. We excluded studies for the following reasons: they were non-systematic reviews (20), they were unobtainable (3), participants did not have stroke at baseline (75), they did not measure blood IL-6 levels (12), death or disability was not reported (20), they reported odds ratios for the association of IL-6 above and below a threshold (2) [15],[16], they reported correlation coefficients only (4) [17]–[20], they reported mean levels in patients with good and bad outcomes only (5) [21]–[25], or they did not report numerical results (1) [26]. We identified four relevant studies(Table 5) [27]–[30] that yielded, for the association between IL-6 and poor outcome, 1,037 patients, and IL-6 and death 1,122 patients. The summary odds ratios were comparable to the results of the current study (Figure 4).

**Table 5:** Table of studies included in the systematic review.

Study	Stroke Diagnosis	Markers Measured	Blinding of Marker Measurement to Stroke Status	Defined Enrolment Period	Adequate Description of Marker Measurement	Mean Age	Male %	Outcome	Number of Patients (Number With Poor Outcome)	Covariates in Model
Blanco 2006 [27]	Clinical supported by imaging	IL-6, L-arginine, TNF, Glutamate, GABA, Fibrinogen	?	Yes	Yes	70	58	Poor outcome 3 mo	113 (36)	HBP, Age, SBP, Temp, Glucose, CSS, Arginine
Welsh 2009 [30]	Clinical supported by imaging	IL-6, CRP, IL-18, TNF alpha, D dimer	?	Yes	Yes	69	53	Poor outcome 1 mo	219 (94)	Age, OCSP, SSS score, CRP, IL-18, TNF
Chamorro 2007 [28]	Clinical supported by imaging	IL-6, normetanephrines	?	No	No	74	43	Death 3 mo	136 (16)	NIHSS, Infection, Neutrophils, Monocytes, Normetanephrines
Rallidis 2006 [29]	Positive imaging only	IL-6, CRP, Serum Amyloid A	?	Yes	Yes	54	65	Death in hospital	203 (14)	Age, Sex, BMI, HBP, Cholesterol, DM, Smoking, CRP, Serum, Amyloid A
Whiteley 2009	Clinical supported by imaging	IL-6, CRP, fibrinogen	Yes	Yes	Yes	72	53	Poor outcome 1 mo	844 (238)	Lives alone, independent prior to stroke, age, able to walk, lift arms, talk

All studies were prospective, inpatient-based studies of patients with ischaemic stroke and drew blood soon after stroke. No previous study examined unselected admissions of patients with stroke.

IL-18, interleukin-18; TNF, tumour necrosis factor alpha; GABA, gamma-amino-butyric acid; SBP, systolic blood pressure; HBP, high blood pressure; CSS, Canadian stroke scale; SSS, Scandinavian stroke scale.

## Discussion

### Statement of Main Findings

In this large cohort of stroke patients, we found that higher levels of IL-6, CRP, and white cell count were independently and significantly associated with poor outcome and death at 6 mo after stroke. The association was independent of stroke severity, age, and risk factors for recurrent stroke, though only IL-6 was independent of other markers. The addition of IL-6 to a validated prognostic model increased the proportion of patients with predicted probabilities of a poor outcome of>90% or<10% by only 2.8%, and the net classification index by 5%. These findings lend support to the hypothesis that the inflammatory response is associated with poor outcome after stroke. However, although the measurement of the inflammatory response assessed with IL-6 improves prediction of poor outcome, in this cohort the degree was so small that the use of these markers in routine practice is unlikely to be helpful to clinicians aiming to predict the outcome of their stroke patients, for example by selecting individuals for aggressive treatment or palliative care.

### Study Limitations and Potential Biases

We did not exclude patients with infection from the study, a potential confounder as infection after stroke is associated both with higher levels of inflammatory markers and with poor outcome after stroke independently of other factors, as we sought external validity to determine the role of markers in a clinical setting. However, the delay between blood taking and stroke did leave time for the development of complications in some of the more severely affected stroke patients, so a rise in inflammatory markers due to infection rather than brain damage due to stroke may have been responsible for at least part of the observed association. The cohort, consisting of a mixture of outpatients and hospital inpatients, contained relatively mild stroke patients, so models generated from the whole cohort may not be applicable to cohorts containing only patients with severe strokes, as our models may have a ceiling effect at higher stroke severities. We were limited in our ability to recruit more patients with very severe strokes chiefly because of practical barriers to blood taking and informed consent. We dichotomised the Oxford Handicap Scale, measured by postal questionnaire, into “independent” and “dependent.” Although crude, this measure has both internal and external validity [31].

We assessed inflammatory marker levels only at the time of assessment. While serial measurement might have provided more information, a single measurement was strongly associated with outcome but still did not much add to existing prognostic models. It seems unlikely that the additional trouble of obtaining serial samples will be outweighed by additional predictive power.

The use of AUC to choose between predictive models is a subject of some controversy. The AUC analysis is based on rank comparison, which may be problematic for populations in whom the risk of an event is very low (e.g., incident stroke in asymptomatic cohorts) [32]. However, as the risk of poor outcome after stroke is high in this cohort (32%), the use of the AUC seems reasonable. While in this study IL-6 has an association with poor outcome, an extremely strong and independent association needs to be demonstrated before a marker meaningfully improves classification accuracy [33]. We assessed the additional predictive utility of IL-6 with risk stratification tables applying cut points for predicted outcome that are relevant for stroke practice for the treatments that are currently available. Less stringent thresholds of risk could be examined, though it is hard to see how they would be useful in making decisions about individual patients. We have not demonstrated that IL-6 improves prediction in our cohort using our chosen thresholds. Our conclusions would be strengthened by replication of our findings in a validation dataset.

The systematic review is limited in scope, as several other studies relevant to the association between IL-6 and death or poor outcome reported their results either as a comparison of odds of poor outcome above and below optimised cut points or as correlation coefficients; hence, extraction of data per unit increase in marker level was not possible.

### Interpretation

We have demonstrated that blood markers of the acute inflammatory response, in particular IL-6, are associated with death and poor outcome after stroke. The results from this study are broadly comparable to other studies of IL-6 and poor outcome or death after stroke (Figure 4), which supports the generalisability of the findings.

The strengths of the current study in comparison to other studies merit consideration. It is much larger than previous reports and has used a measure of handicap (the mRS) as well as death to define poor outcome. It has used a validated prognostic model to adjust for confounding by stroke severity, age, and prior dependence and has carefully explored the role of these markers in clinical decision making, which, though often proposed, has not been examined before.

IL-6 is induced by tumour necrosis factor α and IL-1β, and then leads to the releases of CRP, fibrinogen, and cell adhesion molecules, though the cellular origin of IL-6 after stroke is not clear. Whether the higher levels of IL-6 are a bystander to, or a cause of, poor outcome is uncertain. Mice deficient in IL-6 showed similar stroke volume and disability at 24 h as mice with normal IL-6 expression [34], suggesting that it may simply be part of the inflammatory response to stroke and not directly pathogenic. The association of IL-6 with poor outcome has been demonstrated in many conditions such as HIV [35], many cancers [36], and occurrence of vascular disease including stroke [37], making it more plausible that IL-6 is a general marker of disease severity rather than part of numerous disease-specific pathways to poor outcome.

## Conclusions

In this large cohort of stroke patients, blood markers of the acute inflammatory response were associated with poor outcome after stroke, though only IL-6 showed independent association after adjustment for confounding factors, including levels of other markers. In this cohort, the addition of IL-6 to a previously validated prognostic model added to the prediction of outcome, but by an amount that is unlikely to be useful in clinical practice. Whether or not inflammatory markers are useful in prediction of recurrent stroke [38],[39] or other vascular events is a separate question, which requires further study.

## Supporting Information

Text S1 MOOSE checklist.
(0.06 MB DOC)

## Abbreviations

AUC: area under receiver operator curve
CRP: C-reactive protein
IL-6: interleukin-6
mRS: modified Rankin Scale
NIHSS: National Institutes of Health Stroke Scale
NRI: net reclassification improvement
OCSP: Oxford Community Stroke Project
