# Supplementary material for: Inflammatory Markers and Poor Outcome after Stroke: A Prospective Cohort Study and Systematic Review of Interleukin-6
Source: PLoS Med. 2009 Sep 8;6(9):e1000145. doi: 10.1371/journal.pmed.1000145 (PMC2730573; doi:10.1371/journal.pmed.1000145)
Supplement: Text S1 — MOOSE checklist. (0.06 MB DOC) [file pmed.1000145.s001.doc]

**MOOSE Checklist**

# The association between interleukin-6 levels and poor outcome after stroke

Systematic review component of **‘**Inflammatory Markers and Poor Outcome after Stroke: A Prospective Cohort Study and Systematic Review of Interleukin 6’

| **Criteria** | | **Brief description of how the criteria were handled in the review** |
| --- | --- | --- |
| **Reporting of background** | |  |
| √ | Problem definition | The association between interleukin 6 and poor outcome after stroke has been studies several times. We wished to place our cohort study into the context of existing literature. |
| √ | Hypothesis statement | Higher levels of interleukin-6 are associated with poorer outcome after stroke. |
| √ | Description of study outcomes | Poor outcome after stroke (death or disability) or death after stroke. |
| √ | Type of exposure | Blood levels of interleukin-6 |
| √ | Type of study designs used | All studies were eligible, though only prospective studies were found using the search strategy. |
| √ | Study population | (a) reported results for patients with acute stroke (not transient ischemic attack);(b) assayed a venous IL-6 in stroke patients; (c) measured outcome using death, disability or handicap scales and (d) reported results in a manner that allowed calculation of OR for poor outcome or death per unit increase in marker, to allow comparison of measures of association between studies. |
| **Reporting of search strategy should include** | |  |
| √ | Qualifications of searchers | William Whiteley BM BCh |
| √ | Search strategy, including time period included in the synthesis and keywords | Time period: from inception of Pubmed and EMBASE to December 2008  **Search strategy:**  1. cerebrovascular disorders/ or basal ganglia cerebrovascular disease/ or exp brain ischemia/ or carotid artery diseases/ or carotid artery thrombosis/ or carotid stenosis/ or cerebrovascular accident/ or exp brain infarction/ or exp hypoxia-ischemia, brain/ or exp intracranial arterial diseases/ or exp "intracranial embolism and thrombosis"/  2 ((brain or cerebr$ or cerebell$ or vertebrobasil$ or hemispher$ or intracran$ or intracerebral or infratentorial or supratentorial or middle cerebr$ or mca$ or anterior circulation) adj5 (isch?emi$ or infarct$ or thrombo$ or emboli$ or occlus$ or hypoxi$)).tw.  3 (isch?emi$ adj6 (stroke$ or apoplex$ or cerebral vasc$ or cerebrovasc$ or cva or attack$)).tw.  4 1 or 2 or 3  5 exp Interleukins/  6 interleukin 6 or interleukin-6 or il 6 or il6 or il-6.tw  7 5 or 6  8 Incidence/ or exp mortality/ or follow up studies/ or mortality/ or prognos$.tw. or predict$.tw. or course.tw or rankin.tw or Glasgow outcome scale.tw or NIHSS.tw  9 4 and 7 and 8  10 limit 9 to humans |
| √ | Databases and registries searched | PubMed and EMBASE |
| √ | Search software used, name and version, including special features | Ovid was used to search PubMed and EMBASE  Reference Manager 10 used to manage references |
| √ | Use of hand searching | We searched bibliographies of retrieved papers |
| √ | List of citations located and those excluded, including justifications | The literature search identified 146 studies. Of these, studies were excluded for the following reasons: non-systematic reviews (20), they were unobtainable (3), participants did not have stroke at baseline (75), they did not measure blood IL6 (12), death or disability was not reported (20), reported odds ratios for the association of IL6 above and below a threshold (2), reported correlation coefficients only (4), reported mean levels in patients with good and bad outcome only (5) or did not report numerical results (1). A list of retrieved articles is available from the authors. |
| √ | Method of addressing articles published in languages other than English | We placed no restrictions on language; one Chinese and one Georgian article could not be located |
| √ | Method of handling abstracts and unpublished studies | No searching of the grey literature performed |
| √ | Description of any contact with authors | None |
| **Reporting of methods should include** | |  |
| √ | Description of relevance or appropriateness of studies assembled for assessing the hypothesis to be tested | Table of included studies (Table 5) |
| √ | Rationale for the selection and coding of data | We extracted data from logistic regression models reporting the association between interleukin 6 and poor outcome or death after stroke, and the degree of adjustment for age, stroke severity and other potential confounders. |
| √ | Assessment of confounding | The adjustment for confounding in each study is reported, considering the important confounders of the association and stroke outcome to be age and stroke severity. |
| √ | Assessment of study quality, including blinding of quality assessors; stratification or regression on possible predictors of study results | Only 4 studies were available, hence sensitivity analyses were not felt appropriate |
| √ | Assessment of heterogeneity | Heterogeneity of the studies were explored with I^2^ statistic that provides the relative amount of variance of the summary effect due to the between-study heterogeneity. |
| √ | Description of statistical methods in sufficient detail to be replicated | We performed fixed effects meta-analysis with Stats Direct Version 2.7.2 |
| √ | Provision of appropriate tables and graphics | See figure 4 and Table 5 |
| **Reporting of results should include** | |  |
| √ | Graph summarizing individual study estimates and overall estimate | Figure 4 |
| √ | Table giving descriptive information for each study included | Table 5 |
| √ | Results of sensitivity testing | Only 4 studies were available, hence sensitivity analysis not felt appropriate |
| √ | Indication of statistical uncertainty of findings | 95% confidence intervals were presented with all summary estimates, I^2^ values and results of sensitivity analyses |
| **Reporting of discussion should include** | |  |
| √ | Quantitative assessment of bias | The systematic review is limited in scope, as several other studies relevant to the association IL-6 and death or poor outcome reported their results either as a comparison of odds of poor outcome above and below and optimised cut points or as correlation coefficients hence extraction of data per unit increase in marker level was not possible. |
| √ | Justification for exclusion | All studies were excluded based on the pre-defined inclusion criteria. |
| √ | Assessment of quality of included studies | Brief discussion |
| **Reporting of conclusions should include** | |  |
| √ | Consideration of alternative explanations for observed results | The discussion of other reasons for the association between IL6 and poor outcome is made in the discussion of the results of the cohort study. |
| √ | Generalization of the conclusions | Discussed in the context of the results of the cohort study. |
| √ | Guidelines for future research | Whether or not inflammatory markers are useful in prediction of recurrent stroke or other vascular events requires further study. |
| √ | Disclosure of funding source | **Funding/Support:** Dr Whiteley is supported by a Chief Scientist’s Office Clinical Academic Training Fellowship from the Scottish Government. Dr Sudlow was supported by a Clinician Scientist Award from the Wellcome Trust (063668/Z/01/A) and is now funded by the Scottish Funding Council. Ms Jackson was supported by the Wellcome Trust (063668/Z/01/A) and now holds a Binks Trust Research Fellowship.  **Role of the Sponsors:** No funding organization or sponsor had any role in the design and conduct of the study, in the analysis and interpretation of the data, or in the preparation, review, or approval of the manuscript. No author has any other conflict of interest to declare. |
